# Arcuate eminence distance to temporal bone outer table in the middle fossa repair of superior canal dehiscence

**DOI:** 10.1007/s00405-024-09067-0

**Published:** 2024-12-07

**Authors:** Hong-Ho Yang, Isaac Yang, Quinton S. Gopen

**Affiliations:** 1 Department of Head and Neck Surgery, David Geffen School of Medicine, University of California, Los Angeles, 200 UCLA Medical Plaza, Suite 550, Los Angeles, CA 90095 USA; 2 Department of Neurosurgery, David Geffen School of Medicine, University of California, Los Angeles, 200 UCLA Medical Plaza, Suite 550, Los Angeles, CA 90095 USA

**Keywords:** Temporal bone outer table, Superior canal dehiscence, Middle cranial fossa approach, Air–bone gap

## Abstract

**Purpose:**

To investigate the influence of arcuate eminence’s distance to temporal bone outer table (AE-OT) on surgical outcomes following the middle fossa repair of superior canal dehiscence (SCD).

**Methods:**

We conducted a cohort study of consecutive repairs at a center between 2011 and 2022. AE-OT was measured on temporal bone CT imaging. Surgical outcomes were assessed with established metrics including Symptom Resolution Score (SRS), rate of Overall Symptom Improvement (OSI), and change in low-frequency air–bone gap (ΔLF-ABG) from pre- to post-surgery. Multivariable regression models assessing surgical outcomes were constructed with AE-OT as the primary predictor. Models adjusted for patient demographics, medical and surgical history, and follow-up duration.

**Results:**

A total of 402 repairs were included. Mean AE-OT was 27.1 mm (SD 2.1, range 20.8–33.9). Every mm increase in AE-OT was independently associated with a 14% reduction in odds of OSI (aOR 0.86, 95% C.I. [0.75, 0.98]) and a 4-point decrease in SRS (adj. β − 4.0 [− 6.9, − 1.1]) among frank dehiscences. AE-OT was also not associated with operative duration and ΔLF-ABG among both frank dehiscences and near dehiscences.

**Conclusions:**

Longer AE-OT predicted poorer symptomatic response but similar operative duration and audiometric improvement among frank SCD cases.

## Introduction

Superior canal dehiscence syndrome (SCDS) is a rare neurotologic condition caused by incomplete thickening or closure of the bony superior semicircular canal (SCC), resulting in a pathological aperture or thinning of the overlying otic capsule. The presence of this “third mobile window” can alter the acoustic dynamics of the inner ear, resulting in autophony, hyperacusis, and sound- or pressure-induced vertigo [1–3]. Following the presentation of this symptomatology profile, SCDS is confirmed with high-resolution computed tomographic (HRCT) of the temporal bone and objective testing of third-window physiology such as pure tone audiometry and vestibular-evoked myogenic potential (VEMP) testing. Cases with debilitating, persistent symptoms refractory to conservative management can be treated surgically with a middle cranial fossa (MCF) or a transmastoid approach [4–6].

Since surgical repair of SCDS is elective, an important point of deliberation when considering surgery is the probability of success and risks associated with the procedure. Although SCD repair resolves auditory symptoms at high rates, it is feared to precipitate symptoms of imbalance due to potential damage to nearby vestibular organs [7–10]. As symptomatic response can vary widely between patients of different demographics and medical history, identifying pre-operative predictors of surgical outcome is instrumental in guiding the appropriate modality of management [10–12].

A key element in the diagnostic workup of SCDS is HRCT of the temporal bone. The imaging allows for careful analysis of the dehiscence characteristics and anatomical intricacies of its surrounding structures [4–6, 11, 13]. In this study, we focus on a measurement often performed during preoperative surgical planning, the distance between arcuate eminence and the outer table of temporal bone (AE-OT) [14, 15]. AE is an important anatomical landmark of the MCF approach, and AE-OT provides a general overview of the location of the SSC and depth of the craniotomy needed to access the dehiscence [14, 16, 17]. Given its direct surgical relevance, AE-OT may play a role in the outcome of the MCF repair.

Employing a single institutional series of SCD repairs over the past decade, we conduct the first investigation on the relationship between AE-OT and surgical outcomes from the MCF repair of SCD. We hypothesize that longer AE-OT would correlate with increased operative duration because a greater depth of surgical approach would theoretically be required to access the dehiscence. However, we hypothesize a similar degree of symptomatic and audiometric improvement because the dehiscence could be adequately repaired regardless of AE-OT based on our experience.

## Materials and methods

### Study design and population

We conducted a cohort study of consecutive MCF repairs of SCD at a single tertiary institution between 2011 and 2022. Cases must fulfill criteria for a formal diagnosis of SCDS to be considered for surgery. Diagnostic criteria employed are well described by the Barany Society guidelines, including patient symptomatology, HRCT temporal bone, and one form of objective third window physiology testing (e.g., pure tone audiometry, VEMP, etc.) consistent with SCDS [6]. Patients meeting the diagnostic criteria were initially managed conservatively with medical therapy and recommendations of functional measures to avoid provoking stimuli. Cases with debilitating symptoms significantly impairing quality of life and non-responsive to conservative management were offered surgery. The pre-operative HRCT imaging of the temporal bone for all included cases was reviewed to obtain the measurement of AE-OT. Surgical outcomes, assessed with self-reported symptom resolution, audiometric changes following repair, and operative duration, were compared between cases with different AE-OT lengths. Study approval was obtained from the Institutional Review Board at authors’ institution (IRB#22–000259).”

### Surgical technique

The temporal fossa was exposed with a minimally invasive middle fossa craniotomy. The dehiscence was then repaired with bone wax and titanium mesh or autologous bone chip. The sealed dehiscence site was then reinforced with collagen sponges and fibrin glue. All steps of operative repair, from craniotomy to dehiscence closure, for all cases included in this study were performed by the same pair of neurosurgeon and neurotologist employing the same technique.

### Variable characterization

**Fig. 1:**
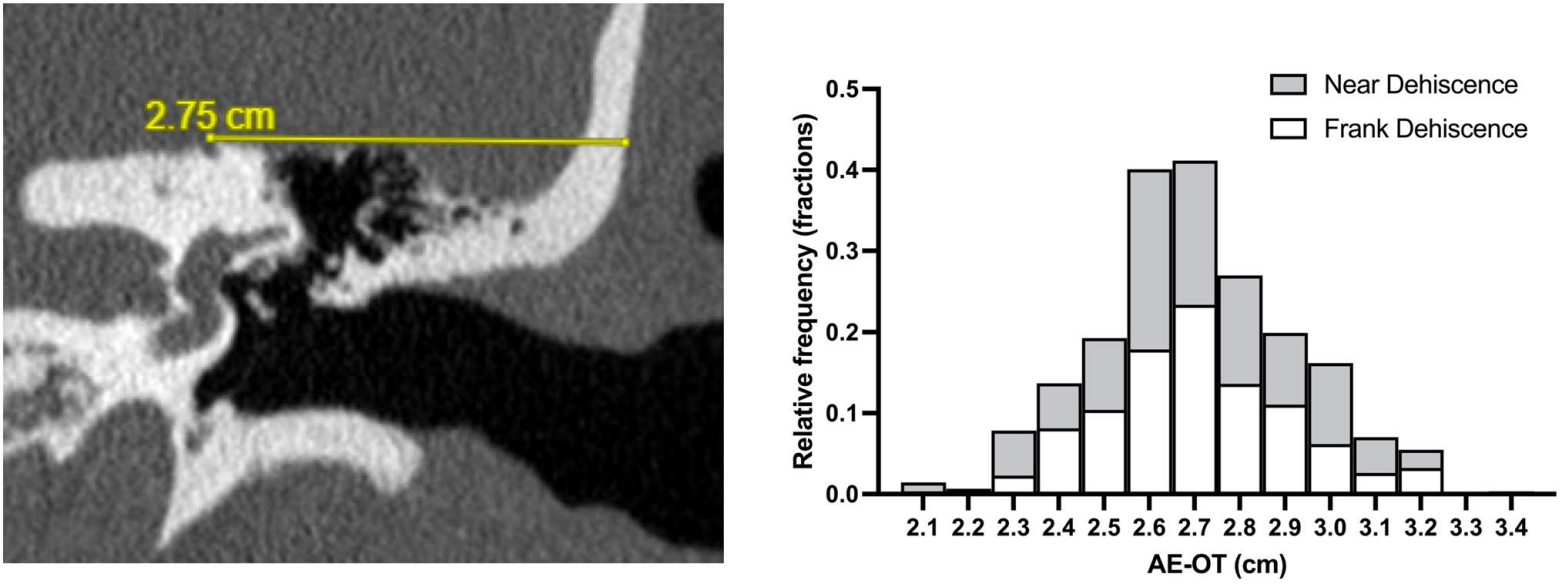
Measurement of the distance between arcuate eminence and temporal bone outer table on computed tomography imaging (left) and distribution histogram of distance between arcuate eminence and outer table of the temporal bone (right)

*Independent variable*: AE-OT measurements (in mm) on coronal view of HRCT temporal for all cases were performed by a single rater blinded to all clinical information, as illustrated in Fig. 1. Measurement protocol was adapted from a prior radiographic review of SCDS cases *(Lookabaugh *et al.*)* [15]. Cases with unique anatomy were reviewed jointly with the operating surgeon alongside neuroradiology input until a consensus was reached.

*Outcome measures*: Surgical outcomes were assessed in three domains: symptomatic response, audiometric outcome, and operative duration. Symptomatic response was assessed with a symptom questionnaire completed by all patients pre- and post-operatively, indicating whether a series of 11 symptoms (autophony, hyperacusis, hearing loss, amplification of internal sounds, tinnitus, ear fullness, dizziness, vertigo, disequilibrium, oscillopsia, headache) were present at the time of completion. Autophony refers to sensitivity to one’s own voice, hyperacusis refers to sensitivity to external stimuli, and amplification refers to sensitivity to other bodily sounds including footsteps, breathing, heartbeat, and digestion. Two previously established metrics of symptom outcomes were computed: Overall Symptom Improvement (OSI) and Symptom Resolution Score (SRS) [9, 11, 12]. OSI evaluates whether the patient experiences net complete resolution of one or more of the most debilitating symptoms. SRS quantifies the net % of preoperative symptoms that resolved following surgery. Audiometric outcomes were assessed with pure tone audiometry administered pre- and post-operatively. Since the audiometric manifestation of SCD is known to be air–bone gap at low-frequencies (LF-ABG), specifically 250-500 Hz, we computed the numerical difference in LF-ABG from pre- to post-MCF. Finally, operative duration was measured as the time between surgical incision and procedure completion (in minutes).

*Control measures*: We also included variables from patient charts that were important to account for in multivariable analyses, including patient age, sex, body mass index (BMI), prior ear surgery status (of the affected ear), American Society of Anesthesiologists (ASA) classification of physical status, dehiscence type (frank vs. near), bilateral SCD disease, and follow-up duration (for symptom questionnaire and audiometry).

### Statistical analysis

Outcome measures were assessed in multivariable regression models with AE-OT as the primary linear predictor (in cm). A logistic regression model for OSI, a bootstrapped linear regression model for SRS, a linear regression model for change in LF-ABG, and a linear regression model for operative duration were constructed. Several iterations of the models were performed to assess the association between AE-OT and surgical outcome as different sets of confounders were adjusted for. Model 1 controls for basic demographic information (age, sex) and follow-up duration. Model 2 controls for patient medical and surgical history (bilateral SCD disease, prior ear surgery, ASA classification) in addition to all variables included in model 1. Finally, as AE-OT can be potentially influenced by head circumference, which is shown to be associated with body height and weight in some cases, model 3 adjusts for BMI in additional to all variables in model 2 [18].

To capture a more granular picture of symptomatic response in addition to OSI and SRS, we separated cases into four cohorts based on individual symptom outcome. Patients reporting the presence of the particular symptom preoperatively reported either resolution (cohort 1) or persistence (cohort 2) following repair. Patients reporting the absence of the particular symptom preoperatively reported either the onset (cohort 3) or continued absence (cohort 4) of that symptom following repair. AE-OT measurements were compared between the four cohorts employing independent sample t-tests, with focus on effect size given limited statistical power due to case stratification. We selected a cutoff of |Cohen d|≥ 0.4 given this is the threshold where both Cohen’s U_3_ and probability of superiority exceed 60%. The primary statistical outcome will focus on OSI and SRS. The secondary analyses on individual symptoms are meant to further contextualize the nature of the findings for OSI and SRS.

Since frank and near dehiscences are distinct in pathophysiology and clinical presentation, we conducted stratified analyses based on dehiscence type [4, 5, 19]. All analyses were performed using Stata, version 17, and Prism GraphPad, Version 10. Statistical significance was assessed at two-tailed, α = 0.05. Interpretations focused on effect size and confidence level, with statistical significance de-emphasized and p-values omitted.

## Results

A summary of study population characteristics is provided in Table 1. Among a total of 402 cases included, average age was 49 years, 63% were females, average BMI was 28 kg/m^2^, and 20% had a prior ear surgery. Most patients had ASA class I or II functional status. 77% of cases were frank dehiscences and 23% were near dehiscences. With a mean symptom follow-up of 4.5 months, 73% of cases achieved OSI and average SRS was 39 (out of 100). With an average audiometric follow-up of 5.3 months, the mean change in LF-ABG from pre- to post-MCF was − 5.2 dB HL. Average operative duration was 1.7 h. A distribution histogram of AE-OT measurements is provided in Fig. 1. AE-OT demonstrated a normal distribution, with and mean and median both at 2.7 cm. Standard deviation was 0.2 cm and mode was 2.7 cm. Most patients had an AE-OT of either 2.6 or 2.7 cm, followed by 2.8 cm, 2.9 cm, and 2.5 cm. Mean AE-OT was 2.70 cm for near dehiscence cases and 2.71 cm for frank dehiscence cases.

**Table 1:** Summary of overall study population characteristics

		AE-OT ≤ 27 mm (N = 201)	AE-OT > 27 mm (N = 201)	Total (n = 402)
Patient history				
	Age, year	48 ± 13	51 ± 11	49 ± 12
	Sex, female	70.1%	56.2%	63.2%
	BMI, kg/m^2^	27 ± 7	28 ± 7	28 ± 7
	Prior ear surgery	23.3%	16.4%	19.9%
ASA classification				
	I	3.5%	5.5%	4.5%
	II	72.6%	67.7%	70.1%
	III	23.9%	26.9%	25.4%
Dehiscence type				
	Frank	75.6%	79.1%	77.4%
	Near	24.4%	20.9%	22.6%
Follow-up duration, month				
	Symptom Questionnaire	4.7 ± 6.5	4.3 ± 6.5	4.5 ± 6.5
	Audiometry	6.1 ± 7.7	4.6 ± 6.5	5.3 ± 7.1

**Fig. 2:**
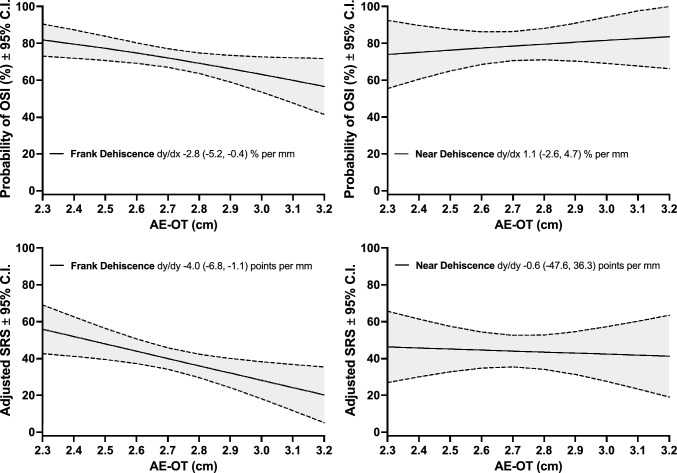
Adjusted regression estimates of Overall Symptom Improvement (OSI) rate and Symptom Resolution Score (SRS) for frank dehiscence (left) and near dehiscence (right) as a function of the distance between arcuate eminence and outer table of temporal bone (AE-OT). All regression estimates are computed from model 3. Legends depict the slope of regressions and 95% confidence intervals

Holding the effect of all potential confounders constant, each centimeter increase in AE-OT was associated with a 14% reduction in odds of OSI (aOR 0.86, 95% CI 0.75–0.98) and a nearly 4-point decrease in SRS (Adjusted β − 4.0, 95% CI − 6.9 to − 1.1) among frank dehiscences but was not significantly associated with odds of OSI or SRS among near dehiscences, Table 2. Among frank dehiscences, the adjusted predicted probability of OSI drops from 81.8% (95% CI 73.0–90.5) at 2.3 cm AE-OT to 56.6% (41.4–71.7) at 3.2 cm AE-OT, and the adjusted predicted SRS drops from 56 (42.7–69.1) at 2.3 cm AE-OT to 20 (5.1–35.3) at 3.2 cm AE-OT, Fig. 2. AE-OT was not significantly associated with the degree of change in LF-ABG from pre- to post-MCF. In the crude model, longer AE-OT was associated with a greater reduction in LF-ABG following surgery (aβ − 1.9, 95% CI − 3.4 to − 0.3 per mm) among near dehiscences. However, this association was no longer present when adjusting for patient demographics and follow-up period, Table 2. Our analyses showed a statistically non-significant trend of decreased operative duration among cases with longer AE-OT.

**Table 2:** Regression modeling, distance between arcuate eminence and outer table of the temporal bone (AE-OT) and surgical outcomes

	Regression outcome	Crude model	Model 1^a^	Model 2^b^	Model 3^c^
Frank Dehiscence	**Symptom**	β or Odds Ratio (95% C.I.) – per mm increase in AE-OT			
	SRS (linear)	** − 3.6 (− 6.4, − 0.9)**	** − 4.2 (− 7.0, − 1.3)**	** − 4.2 (− 7.0, − 1.4)**	** − 4.0 (− 6.9, − 1.1)**
	OSI (logistic)	**0.88 (0.78, 0.99)**	**0.86 (0.76, 0.97)**	**0.85 (0.75, 0.96)**	**0.86 (0.75, 0.98)**
	**Audiometry**	β (95% C.I.) – per mm increase in AE-OT			
	ΔLF-ABG, dB	− 0.3 (− 1.2, 0.5)	− 0.3 (− 1.2, 0.6)	− 0.2 (− 1.1, 0.8)	− 0.2 (− 1.2, 0.8)
	**Operative duration**	β (95% C.I.) – per mm increase in AE-OT			
	Surgery length, hour	− 0.02 (− 0.06, 0.01)	− 0.03 (− 0.06, 0.01)	− 0.03 (− 0.06, 0.01)	− 0.03 (− 0.06, 0.004)
Near Dehiscence	**Symptom**	β or Odds Ratio (95% C.I.) – per mm increase in AE-OT			
	SRS (linear)	0.3 (− 3.5, 4.0)	− 1.0 (− 4.8, 2.9)	− 0.9 (− 4.7, 3.0)	− 0.6 (− 4.8, 3.6)
	OSI (logistic)	1.12 (0.89, 1.41)	1.07 (0.84, 1.37)	1.09 (0.85, 1.41)	1.09 (0.81, 1.46)
	**Audiometry**	β (95% C.I.) – per mm increase in AE-OT			
	ΔLF-ABG, dB	** − 1.9 (− 3.4, − 0.3)**	− 1.2 (− 2.9, 0.4)	− 1.1 (− 2.9, 0.6)	− 1.3 (− 3.1, 0.5)
	**Operative duration**	β (95% C.I.) – per mm increase in AE-OT			
	Surgery length, hour	− 0.04 (− 0.1, 0.02)	− 0.03 (− 0.09, 0.03)	− 0.03 (− 0.01, 0.03)	− 0.05 (− 0.1, 0.02)

*SRS*, symptom resolution score, *OSI* overall symptom improvement, *LF-ABG* low-frequency air–bone gap

Bold values, CI does not overlap the null

Positive* β* indicates increased score from pre- to post-op; negative *β* indicates decreased score from pre- to post-op

^a^Model 1 adjusts for patient age, sex, and follow-up duration

^b^Model 2 adjusts for bilateral SCD disease, prior ear surgery, and ASA status in addition to all covariates in Model 1

^c^Model 3 adjusts for body mass index in addition to all covariates in Model 2

**Fig. 3:**
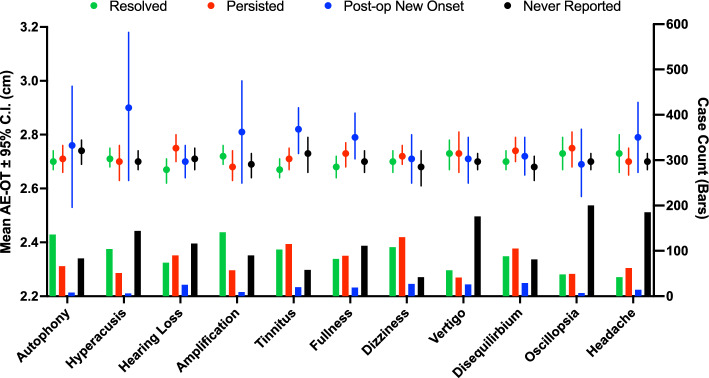
Distance between arcuate eminence and outer table of the temporal bone (AE-OT) for frank dehiscence cases reporting the presence of the particular symptom preoperatively which resolved (green) or persisted (red) following surgery, and cases reporting the absence of the particular symptom preoperatively which experienced symptom onset postoperatively (blue) or did not experience symptom onset postoperatively (black)

Further analyses of the outcomes of individual symptoms are provided in Fig. 3. The mean AE-OT of cases with resolution of hearing loss was shorter than the mean AE-OT of cases without resolution of hearing loss (2.67 vs. 2.75 cm, Cohen d − 0.4). The mean AE-OT of cases that reported postoperative onset hyperacusis (2.90 vs. 2.70 cm, Cohen-d 0.9), amplification of internal sounds (2.81 vs. 2.69 cm, Cohen-d 0.5), tinnitus (2.81 vs. 2.73 cm, Cohen-d 0.4), ear fullness (2.79 vs. 2.70, Cohen-d 0.4), and headache (2.79 vs. 2.70 cm, Cohen-d 0.5) was higher than the mean AE-OT of cases that did not.

## Discussion

In this cohort study of 402 SCD repairs via the MCF approach, we found longer AE-OT to associate with poorer symptomatic response among frank dehiscence cases despite similar operative duration and audiometric improvement. Namely, patients with longer AE-OT reported lower resolution rates of hearing loss and higher postoperative onset rates of hyperacusis, tinnitus, ear fullness, and headache.

A 2007 anatomic study (*Djalilian *et al.) on the middle cranial fossa of 98 non-SCDS temporal bones found the average distance between SSC dome and temporal bone outer table (SSC-OT) to be 2.1 cm [14]. More recently, a 2014 radiographic review of 202 SCDS patients (*Lookabaugh *et al*.*) found the mean distance of AE-OT to be 2.6 cm [15]. We add to the literature with the largest cohort, demonstrating a mean AE-OT of 2.7 cm among 402 SCD cases, which is similar to findings from *Lookabaugh *et al. Although AE is not always near the SSC dome, *Lookabaugh *et al. approximates the location of AE near the SSC dome.[14, 15]. Therefore, the measurements of SSC-OT by *Djalilian *et al. and the approximate measurements of AE-OT by *Lookabaugh *et al. should be comparable. However, a noticeable discrepancy emerges: the mean SSC-OT among non-SCDS cases in *Djalilian *et al*.* is markedly lower than the mean AE-OT among SCDS patients in both *Lookabaugh *et al*.* and the present study. As such, the AE-OT of SCDS patients may be longer compared to that of non-SCDS patients, although direct comparative studies are needed to ascertain this relationship.

Poorer temporal bone pneumatization is closely associated with the pathogenesis of SCDS [20, 21]. Given the observation of longer AE-OT among SCD cases, we surmise that AE-OT may implicate structural and congenital factors related to temporal bone pneumatization. We speculate that better-pneumatized mastoids may be associated with shorter AE-OT due to a more vertical and less horizontal, medially sloping orientation [22, 23]. However, this theory is mostly conjectural as very limited evidence on the clinical implications of AE-OT exists in the literature. To date, poorer temporal bone pneumatization has only been correlated with shorter mastoid height and shorter distance of jugular bulb and sigmoid sinus to internal acoustic canal, external acoustic canal, and the middle ear [24, 25].

The present study is the first to investigate the influence of AE-OT on surgical outcomes following the MCF approach. Our analyses revealed that longer AE-OT was associated with poorer symptom outcomes despite similar audiometric improvement among frank dehiscences. Specifically, the association of longer AE-OT with lower rates of OSI and lower SRS was primarily driven by lower resolution rates of hearing loss and higher postoperative onset rates of hyperacusis, amplification of internal sounds, tinnitus, ear fullness, and headache. Since the poorer symptomatic response was primarily due to the postoperative onset of symptoms rather than the lack of resolution of preoperative symptoms, we do not believe the underlying mechanism is related to inadequate repair of SCD. Furthermore, the LF-ABG effectively narrowed, and the classic auditory symptoms of third window physiology resolved at high rates regardless of AE-OT. Therefore, we surmise that the underlying mechanism may be more related to patients’ inherent anatomy, physiology, and embryology.

Important confounders that may influence AE-OT include head circumference and intracranial hypertension [26]. Patient sex may also be an important factor because males are known to have higher head circumference than females [27]. However, our analyses found the association between AE-OT and symptom outcomes to persist even after controlling for patient sex and BMI, a marker of both head circumference and intracranial hypertension. Therefore, we believe the association may be better explained by the structure and developmental intricacies of the temporal bone.

If longer AE-OT is indeed associated with poorer temporal bone pneumatization as we previously hypothesized, then cases with longer AE-OT would be expected to report less favorable symptom outcomes. Previous research has demonstrated the protective and “shock-absorbing” effect of pneumatization against trauma to the temporal bone. Specifically, studies have highlighted the association of greater pneumatization with reduced incidence of otic-capsule violation and less severe hearing impairment from temporal bone fractures [28, 29]. Increased pneumatization of the temporal bone was also shown to yield better hearing outcomes from pediatric tympanoplasty [30]. Therefore, patients with less adequately pneumatized temporal bones may be expected to be less resistant to hearing loss following the MCF approach, which may explain the lower resolution rate of hearing loss among cases with longer AE-OT.

Furthermore, the relationship between AE-OT and functional outcomes may be influenced by side effects related to temporal bone elevation. AE-OT might affect the extent to which the temporal lobe needs to be elevated, with longer AE-OT potentially necessitating a greater degree of elevation and consequently a higher risk of central nervous system side effects. This increased risk may lead to more pronounced auditory symptoms, as highlighted in this study.

Our series included very few patients with postoperative onset hyperacusis/amplification (n < 10), and these patients had longer AE-OTs (mean 2.9 cm) compared to patients without postoperative onset hyperacusis/amplification. Given the association between longer AE-OT and SCDS, the temporal bone of patients with longer AE-OT may also be less dense and insulating against acoustic energy. As such, they would be at higher risk for hyperacusis, especially after a traumatic event or an invasive procedure such as the MCF. Temporal bones that are less dense may also require a longer course of recovery following craniotomy, which may be responsible for the prolonged onset of non-specific symptoms following repair (tinnitus, fullness, and headache) among patients with longer AE-OTs. It is also possible that frank dehiscences in patients with longer AE-OT are associated with higher rates of near dehiscence in the neighboring regions of the otic capsule, hence the postoperative onset hyperacusis [31]. Ultimately, additional research on the association of AE-OT with the density and pneumatization of temporal bone is warranted. Nevertheless, surgeons should be aware of the implications of AE-OT and patients should be counseled accordingly.

### Limitations

Since AE-OT is neither randomizable nor manipulatable, a cohort study design would be the highest level of evidence possible, and causal inference is thus not permitted. Additionally, VEMP testing was not routinely obtained post-operatively, preventing its inclusion as a reliable measure of objective vestibular outcomes. Further investigation into the relationship between AE-OT and objective vestibular testing (VOR/vHIT, VEMP), both pre- and post-operatively, is needed. Therefore, ongoing research is needed to further characterize the mechanism through which AE-OT influences patient outcomes. Despite these limitations, we present the first analysis of the relationship between AE-OT and functional outcomes employing the largest cohort of SCD surgeries to date. Findings from this study are worthy of deliberation among the neurotology community as part of the ongoing efforts to improve the clinical outcomes of SCDS patients.

## Conclusions

AE-OT is important for preoperative surgical planning and may implicate functional outcomes from the MCF repair of SCD. Patients with longer AE-OT experienced the resolution of most symptoms at similar rates and demonstrated a similar degree of audiometric improvement compared to patients with shorter AE-OT. However, they are more likely to report persistent hearing loss and postoperative onset of hyperacusis, tinnitus, ear fullness, and headache. Additional investigation on the potential relationship between AE-OT and temporal bone pneumatization will provide additional insights regarding the highlighted association.

## Data Availability

Data for this study are not publicly available to ensure the protection of patient confidentiality.
